# Pain management after major surgery in elderly patients

**DOI:** 10.1186/1471-2482-13-S1-A11

**Published:** 2013-09-16

**Authors:** Danzi Michele, Servillo Giuseppe, Pannullo Mario, Palumbo Chiara, Reggio Stefano, Grimaldi Luciano

**Affiliations:** 1 Department of Specialized Surgery, Division of Gastrointestinal Surgery Rehabilitation of Election and Emergency, “Federico II” University, Naples, Italy; 2 Department of Surgical Sciences, Anesthesiology, Intensive Care and Emergency. “Federico II” University, Naples, Italy

## Background

After-surgery pain is an extremely variable clinical condition, in a continuously changing evolution, where environmental and personal factors have combinations and results not easily predictable. It puts the subject into a state of physical and mental subjection so as of powerlessness. It could be feared more than the surgery itself and anesthesia. Such fears can bring the patient to delay surgery, increasing its risks, scope and post-surgery pain itself: this may lead to a progression of the pathological condition, and make recovery and convalescence more complicate [1].

After-surgery pain treatment allows to: limit neurovegetative storms and the states of neuroendocrine activation; avoid, as much as possible, the development of "persistent pain" situations; facilitate the re-establishment of the previous psyco-physical function and functional state, so improving the general "patient satisfaction".

The results of recent case studies have shown that after surgery pain management is insufficient; this implies negative economic consequences to the National Health Service, including the increase of unsatisfied patients number, longer hospitalization and frequent re-hospitalizations [5].

The aim of our study is to compare the analgesic effect Ketorolac + Tramadole vs the analgesic effect of Meperidine only, in pain management after Major Surgery. We also focused our attention on the possible occurrence of adverse reactions, identified as every effect occurring within 48 hours after surgery (nausea, vomit, headache, vertigo).

## Methods

The study population consists of 70 patients (Table 1) referred to the Department of Gastrointestinal Surgery of Naples University “Federico II”, who underwent to gastrointestinal resection surgery. Patients aged 65 to 86 were included, with ASA II-III, not subjected to pre-surgery pain therapy. We divided the patients in two groups: M group (Meperidine) and T group (Tramadole + ketorolac), homogeneous for age, gender, type of surgery, anesthesy risk, previous clinical and pathological features, to whom were administered different after-surgery analgesic therapies.

**Table 1:** 

M GROUP (35 patients)	T GROUP (35 patients)
60 ml (2 ml/h) Elastomeric pump	60 ml (2 ml/h) Elastomeric pump
Meperidine 200 mg	Tramadole 100 mg
+Metoclopramide monohydrocloride 10 mg	+Ketorolac 90 mg
+Ranitidine 150 mg	+Metoclopramide monohydrocloride 10 mg
+ saline 39 ml	+Ranitidine 150 mg
	+ saline 38 ml

At the end of surgery each patient had the elastomer according to the protocol and was followed in the next three days, monitoring the possible occurrence of adverse reactions. In both groups, the elastomeric pump was reloaded the day after surgery.

We assessed after surgery pain using the Visual Analogue Scale (VAS); this scale consists of a straight line (100 mm long) representing an intensity sequence between the two ends: "no pain" and "the strongest pain imaginable".

The record of pain intensity was written on the "Pain chart" every three hours and suspended when VAS was equal or lower than three, without any treatment, after three registrations in a row.

## Results

**Figure 1:**
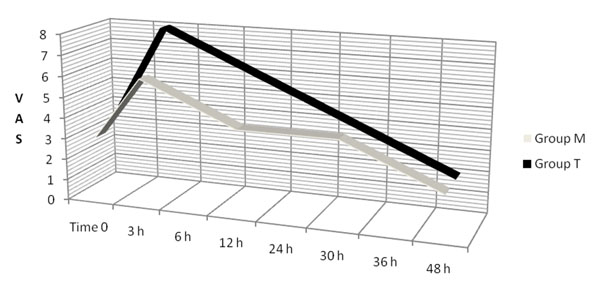


As shown in the graph (figure 1), VAS has a lower value for M group on day one, with respect to T group. Then the data of the two groups align up to overlapping in the following hours.

After two days of observation we suspended the treatment with the elastomeric pump and monitored the patient every 3 h, reporting VAS on the "pain chart" and preparing for the eventual administration, in case of need, of 1000 mg of paracetamol, which occurred in 12 patients, 5 of which were in M group.

Three cases of nausea were recorded in T group vs 12 cases in M group, 2 of which with connected vomit.

After surgery, intestinal gas emission was slower in M group, with an average delay of 12h with respect to T group.

## Conclusions

Our study demonstrates that the two treatments can overlap as for the long time analgesic effect, while it puts into evidence that whenever is recommended a fast recovery of an effective intestinal propulsion, it is of particular disturbance the constipating action of opioids; in fact the extended use of intravenous Meperidine caused, in some patients, intestinal function problems. On the contrary, Tramadole shows a less constipating effect.

Moreover, multimodal analgesia is strongly recommended in after-surgery pain management since it can potentially improve the analgesic effect by affecting various mechanisms and/or by reducing each single agent dose, so minimizing any dose - depending adverse effec as well [4]. It is our opinion that the FANS have still a basic role as supporting drugs (not life-saving) to the therapies aimed to reduce the amount of intravenous opioids administered [3].

The experience of acute pain, as isolated event in someone's life, is a kind of experience that does not imply psychological and behavioral adaptations but, on the contrary, it is connected to an individual reactivity and related to the surgical situation which often implies high levels of psychological distress, since it causes a detrimental action to the individual's body. After surgery analgesic therapy and pain management during the entire patient hospitalization are fundamental as part of the recovery process [2].
